# Complete Genome Sequence of the Human Herpesvirus 6A Strain AJ from Africa Resembles Strain GS from North America

**DOI:** 10.1128/genomeA.01498-14

**Published:** 2015-02-12

**Authors:** J. Tweedy, M. A. Spyrou, C. D. Donaldson, D. Depledge, J. Breuer, U. A. Gompels

**Affiliations:** a Department of Pathogen Molecular Biology, London School of Hygiene and Tropical Medicine, University of London, London, United Kingdom; b MRC/UCL Centre for Medical Molecular Virology, Division of Infection and Immunity, University College London, London, United Kingdom

## Abstract

The genome sequence of human herpesvirus 6A (HHV-6A) strain AJ was determined in a comparison of target enrichment and long-range PCR using next-generation sequencing methodologies. The analyses show 85 predicted open reading frames (ORFs), conservation with sequenced HHV-6A reference strain U1102, and closest identity to the recently determined GS strain, despite different geographic origins (United States and Gambia).

## GENOME ANNOUNCEMENT

The *Roseolovirus* genus of the human *Betaherpesvirinae* subfamily comprises three members: human herpesvirus 6A (HHV-6A), the prototype, HHV-6B, and HHV-7. These viruses are common causes of infant fever, occasionally with rash (then termed exanthema subitum/roseola infantum) (1–3). The infections persist for life and are generally self-limiting, but they are also associated with central nervous system disease. HHV-6A has evidence for increased neurotropism, particularly in glial cells, and is identified in glioma and multiple sclerosis patient subsets (4–8). While HHV-6A infant infections are rare in Europe and North America, they have been observed in Africa (9) and are prevalent to a similar extent as HHV-6B in the recently defined germline chromosomally integrated form, ciHHV-6, in which virus genes might be expressed in every cell (10). Therefore, the drivers of HHV-6A distribution and evolution might be fundamentally different, and knowledge of the viral genomes is needed to understand these relationships.

To date, HHV-6A isolates and complete genome sequences are scarce, with those of only two strains identified, the NCBI reference 159-kbp strain U1102, from a Ugandan HIV/AIDS patient (11, 12), and the 157-kbp strain GS, from American patients with lymphoproliferative disease (13, 14) (GenBank/EBI accession no. X83413.1 [U1102] and KC465951.1 and KJ123690.1 [GS]). We report here the determination of a third HHV-6A strain, AJ, from an adult HIV/AIDS patient from Gambia (15).

We compared the amplification methods required to characterize strains directly from infected tissue, using both Agilent SureSelect target enrichment (16) and in-house long-range PCR methodologies to generate Illumina sequence libraries from infected-cell DNA. These were paired-end sequenced on an Illumina MiSeq, and a VelvetOptimiser, Velvet (17), and ABACAS (18) pipeline was used to optimize the *de novo* assembly. Both methodologies generated identical consensus sequences with similar variant-calling efficacy. Gaps, ambiguities, and repetitive regions were confirmed by PCR amplification and Sanger sequencing. Repetitive sequences at the genome ends were resolved utilizing the direct repeat structure of the termini and corresponding regions in the opposite termini for the first 715 and final 1,381 bp of direct repeat left (DR_L_) and right (DR_R_). Annotation was generated using the Rapid Annotation Transfer Tool (RATT) (19) with the reference HHV-6A strain U1102 (12) and updates based on GeneMark predictions (20) as well as other subsequently sequenced HHV-6A and HHV-7 strains (13, 21, 22).

The HHV-6A AJ genome is 156,714 bp in length, maintaining a typical class A herpesviral genomic organization consisting of a 140,401-bp unique long region flanked by 8,156-bp direct repeats (DR_R_ and DR_L_). The DRs are bounded by DNA packaging, *pac1* and *pac2* sites, and human telomeric repeats, as shown previously for U1102 (23). Phylogenetic analyses showed the closest relationship to be with the North American isolate HHV-6A strain GS, at 99.1%, followed by HHV-6A strain U1102, at 98.4%. Single-nucleotide polymorphisms (SNPs) are found across the genome, and they are increased in DRs with small indels. Of note, both the AJ and GS strains show DNA polymerase gene (U38) sequence variation confounding the commonly used PCR-based methods for HHV-6A diagnostics (24). There are 85 genes, as shown previously, including analyses of HHV-6A GS, plus HHV-7, a distant roseolovirus (13, 21, 22). Even with distinct geographic origins, the HHV-6A strains AJ and GS are closely conserved, which may reflect highly evolved viral status or recent emergence.

### Nucleotide sequence accession number.

The whole HHV-6A strain AJ genome sequence has been deposited in GenBank under the accession no. KP257584.
